# The Mice Drawer System (MDS) Experiment and the Space Endurance Record-Breaking Mice

**DOI:** 10.1371/journal.pone.0032243

**Published:** 2012-05-29

**Authors:** Ranieri Cancedda, Yi Liu, Alessandra Ruggiu, Sara Tavella, Roberta Biticchi, Daniela Santucci, Silvia Schwartz, Paolo Ciparelli, Giancarlo Falcetti, Chiara Tenconi, Vittorio Cotronei, Salvatore Pignataro

**Affiliations:** 1 Universita’ degli Studi di Genova & Istituto Nazionale per la Ricerca sul Cancro, Genova, Italy; 2 Istituto Superiore di Sanita’, Behavioural Neurosciences Section, Department of Cell Biology and Neurosciences, Roma, Italy; 3 Thales Alenia Space, Vimodrone (MI), Italy; 4 ASI - Agenzia Spaziale Italiana, Roma, Italy; Pennington Biomedical Research Center, United States of America

## Abstract

The Italian Space Agency, in line with its scientific strategies and the National Utilization Plan for the International Space Station (ISS), contracted Thales Alenia Space Italia to design and build a spaceflight payload for rodent research on ISS: the Mice Drawer System (MDS). The payload, to be integrated inside the Space Shuttle middeck during transportation and inside the Express Rack in the ISS during experiment execution, was designed to function autonomously for more than 3 months and to involve crew only for maintenance activities. In its first mission, three wild type (Wt) and three transgenic male mice over-expressing pleiotrophin under the control of a bone-specific promoter (PTN-Tg) were housed in the MDS. At the time of launch, animals were 2-months old. MDS reached the ISS on board of Shuttle Discovery Flight 17A/STS-128 on August 28^th^, 2009. MDS returned to Earth on November 27^th^, 2009 with Shuttle Atlantis Flight ULF3/STS-129 after 91 days, performing the longest permanence of mice in space. Unfortunately, during the MDS mission, one PTN-Tg and two Wt mice died due to health status or payload-related reasons. The remaining mice showed a normal behavior throughout the experiment and appeared in excellent health conditions at landing. During the experiment, the mice health conditions and their water and food consumption were daily checked. Upon landing mice were sacrificed, blood parameters measured and tissues dissected for subsequent analysis. To obtain as much information as possible on microgravity-induced tissue modifications, we organized a Tissue Sharing Program: 20 research groups from 6 countries participated. In order to distinguish between possible effects of the MDS housing conditions and effects due to the near-zero gravity environment, a ground replica of the flight experiment was performed at the University of Genova. Control tissues were collected also from mice maintained on Earth in standard vivarium cages.

## Introduction

### Effect of Microgravity on the Human Body

Humans, like other living organisms on the planet Earth, have evolved by adapting their body biological structures and functions to the gravitational field of Earth. Weightlessness (or *zero-g*) is the condition that exists when a body experiences only the acceleration that defines its inertial trajectory, or the trajectory of a pure free-fall. The space weightless environment affects about every function of the human body. Indeed, space adaptation involves some very complex changes in the human tissues and organs. Although these changes can cause serious health problems both in space and on return to Earth, especially with increased space permanence, in most cases, their effects are only temporarily disabling and, after their return to Earth, astronauts normally re-adapt quickly to the Earth’s gravity environment [1]. The longest duration mission up to date is the one by Valeri Polyakov a Russian astronaut who lived for 438 days on the Mir Space Station in the 90’s [2]. Today, the ISS astronauts are the main target of the studies about the microgravity effects on human body in light of the forecasted long duration future space flights, as a trip to Mars could be. The observed effects of the space environment on the human body are numerous: the first that become evident is the one on blood circulation. On Earth, the heart distributes blood throughout the body and gravity helps to drawn blood to the lower limbs. In space, the heart is still programmed to work as on Earth but the force to pull body fluids down is missing. As result the zero-gravity condition causes in the astronauts a characteristic facial edema, whereas legs became thinner because blood can only be pumped there by the heart with no help from gravity [3]. Some studies showed a decrease in heart rate in rats [4] as well as in humans [5]. Other reported effects on blood circulation include a decreased plasma volume [6], [7] followed by a post-flight hypovolemia [8] and a post-flight postural hypotension [5]. Bone tissue is a structure designed in response to mechanical stimuli and it can adapt during life toward more efficient mechanical performances. Space exposure can alter the delicate balance of homeostasis of weight-bearing bones in normal Earth gravity environment [9], [10] and be the cause of a spaceflight osteopenia [11]. It has been reported that after a 180-day spaceflight the principal bone formation parameters were decreased while bone resorption markers were increased [12] especially in weight-bearing bones [9], [10], [12]. Further studies showed that microgravity caused an increase in osteoclast-mediated resorption of bone resulting in an increased osteoblast apoptosis [11]. Skeletal muscle is another tissue strongly affected by microgravity. In 2008 Tesch and coworkers found that, even after a short time in orbit, a reduction of muscle function was already detectable and that the longer the mission time was extended, the more the muscle function worsened. Indeed, the fibers within the muscle itself were affected [13]. Antigravity muscles, such as soleus and gastrocnemius quadriceps, as well as extensor muscles [14]–[16], are the most affected by the space permanence. Furthermore, the near-zero gravity environment has been reported to affect gene transcription and protein translation in the muscle cells [14], [15]. Systemic factors, such as space related altered hormone production and metabolic changes also contribute to the muscle mass loss. An important consequence of the microgravity exposure detectable during the post-flight period is an increased muscle fatigue. Persistent fiber necrosis, interstitial edema, and activation of macrophages have been observed even weeks after landing [15]. Spaceflight surely has an impact on more than cardio-circulation, bone and muscle physiological functions. For example, one should also consider the risk bound to the observed deterioration of the immune system resulting in a secondary immunodeficiency that can lead to increased infections or autoimmunity [17]. Further studies on the immune system performed by Gridley and colleagues showed a reduction of T-lymphocytes of mice after 13 days in space, thus demonstrating that microgravity has a significant effect on T cell distribution, function, and gene expression after a short-term spaceflight. Experiments performed on thymus revealed an effect on the expression of cancer related genes, indicating a possible increase of carcinogenesis [18]. Moreover, the potential for astronauts to develop cancer could be enhanced by alterations in natural killer cell function, since after space flight the number and cytotoxic activity of natural killer cells was reduced [17]. Furthermore, other studies have shown a decrease in the production or function of specific cytokines and chemokines, such as interferon *α*/*β* [19] and tumor necrosis factor alpha (TNF-*α*) [20]. In addition, the impairment of the immune system during spaceflight can also dramatically alter the body ability to repair itself following a cutaneous or intrinsic wounding [21].

During a space flight mission, astronauts do not receive the same stimuli from the surrounding environment as on Earth. The central nervous system processes involved in the development of the astronaut’s sense of direction are misleading and for astronauts it is difficult to adapt to the microgravity environment. The near-zero gravity mainly affects the vestibular apparatus inside the inner ear: this condition usually lead to a deconditioning of motion sensors as well as of the somatosensory system, to an altered orientation perception and to a loss of balance. This disorientation is the main symptom of a temporary disease known as Space Adaptation Syndrome (SAS), the main cause of Space Motion Sickness (SMS). As a matter of fact, SAS is caused by a sensory conflict between inputs from visual and tactile senses and inputs from the vestibular organs [22]. Thus, in order to reorganize the orientation apparatus, some adaptive changes occur in the neural strategies to perceive spatial information. Visual cues and references become fundamental to astronauts since in space “down” is where the astronaut feet are. Other tissues and organs for which alterations due to a microgravity exposure have been reported include: effects on inner ear [23] as well as on the formation of renal stones after spaceflight [24].

### Advantage of Using Mouse Models to Investigate Microgravity Effects

Understanding mechanisms that characterize the adaptation of the human beings to the space environment is one of the main goals of the astrobiology research. Spaceflight research should take advantages of mice as an animal model since this species is the only one allowing the testing of a wide range of wild-type and mutant animals, a relatively large animal number per flight, and a reduced demand on shuttle resources and crew time during the mission. Mice are the vertebrate species most commonly used for scientific research; since their size is approximately 10% of that of a rat, mice can live in a much smaller area and consume a reduced quantity of food, water and oxygen. Moreover, the reduced mass of an adult mouse (about 30 grams) permits the use of a significantly higher number of animals, thus increasing the amount of acquirable data and the statistic significance of the experiments. In addition, the employment of mice results in other practical advantages such as low cost, ease of handling and fast reproduction rate. Given the remarkable similarity between the mouse and the human genome, mice are widely considered the best model of inherited human disease as well as congenital defects. With the advent of the genetic engineering technology, genetically modified mice can be generated and can provide models for a range of human diseases. Due to the different life spans of the two species, results based on a few months of observations in mice could be comparable to results based on many years of observations in humans.

### International Tissue Sharing Program (TSP)

Since the initial conception of the MDS, we realized that sharing tissues of the flight mice could provide data on the effect of long duration spaceflight in multiple physiological systems. Therefore a Tissue Sharing Program (TSP) was established and the MDS experiment was planned to contribute data on microgravity effects on the skeletal, cardiovascular, and immune systems, liver and kidney functions as well as other physiological systems. Thus, a TSP was organized by ASI with contributes from European Space Agency (ESA), National Aeronautics and Space Administration (NASA), Japanese Space Agency (JSA) and the Canadian Space Agency (CSA) in order to ensure the maximal scientific output of the space mission samples over expensive and rare occasioned life science experiments. The program involved 20 research groups from 6 countries all over the world. During the TSP operations every effort was made to harvest as many different samples and types of tissue as possible from the mice in order to obtain the maximum of data from this unique experiment.

## Materials and Methods

### The Mice Drawer System Payload

**Figure 1:**
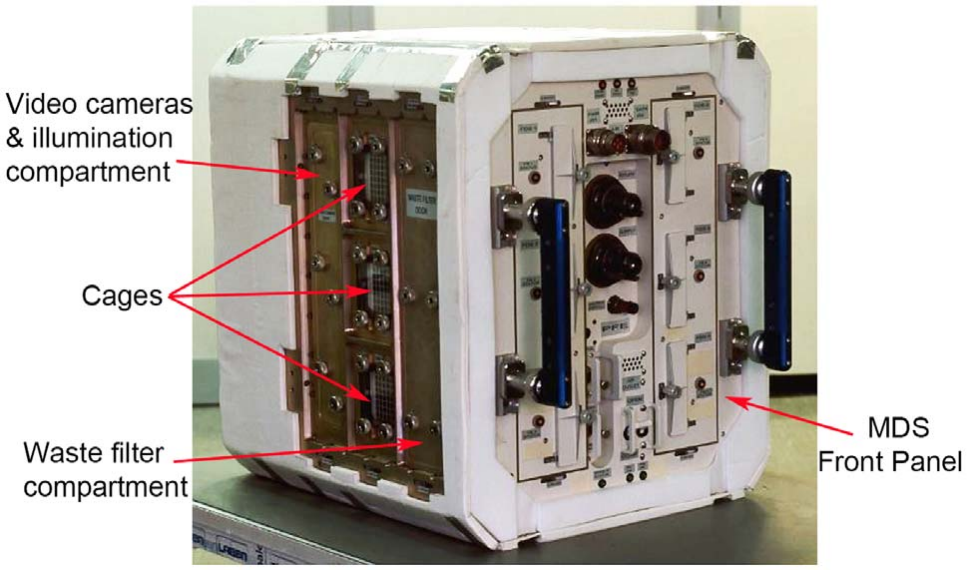
The Mice Drawer System Flight Model.

In 1998 the Italian Space Agency (ASI) Scientific Board selected the MDS program, proposed by the research group at the Genova University, within the frame of the first ASI ISS Utilization Plan. The original scope of the MDS program was to develop a facility for conducting research in microgravity conditions on bone formation and to develop specific countermeasures for osteoporosis. Nevertheless, the MDS payload was developed by Thales Alenia Space – Italia (Milan plant, Italy), a company developing equipment for the support of scientific experimentation in space, as a general purpose facility that could be customized and used in the ISS by a large number of scientists for long duration experiment with mice in different research areas. Indeed, in the standard configuration, MDS can house 6 mice individually up to 100 days with possible extension to 180 days on board the ISS. MDS consists of the following main Subsystems all integrated inside an External Container (516×421×480 mm): the Mice Chamber (MC), the Liquid Handling Subsystem (LHS), the Food Delivery Subsystem (FDS), the Air Conditioning Subsystem (ACS), the Illumination Subsystem (ILS), the Observation Subsystem (OSS) and the Payload Control Unit (PCU) (Figure 1). The Mice Chamber is divided in 2 habitats. Each habitat permits the accommodation of the items necessary to provide 3 individually housed mice with basic services such as three metallic cages each one with a 116×98 mm floor area (configuration flown in the first mission with one mouse/cage), three food envelopes, each one including two food bars, three drinking valves for water delivery, three cameras for video observation, white and infrared LED’s for illumination and sensors for air composition monitoring and control (temperature, rH, CO_2_ and NH_3_). Cages have grids in all four walls, permitting olfactory but not physical contact between animals. According to the scientific requirements, each animal habitat can also be configured as a unique cage of about 364×98 mm floor area (to house a group of mice), or as two cages of about 178×98 mm floor area (to house two pairs of mice) (Figure S1). The Liquid Handling Subsystem delivers drinkable water to each cage individually by means of commercial stem activated drinking valves (made by Edstrom, Waterfors, WI, USA) connected to a Main Water Tank of about 0.5 liters capacity. The water is delivered “ad libitum” (expected max. water consumption is 7 ml/day/mouse). In order to support the whole mission duration, the internal Water Tank must be refilled in orbit once empty (about every 10 days). The Food Delivery System supplies each cage with 2 food bars for a total food mass of about 90 grams. Food bars composition can be defined according to the experiment protocol and, if needed, solid drugs and additives can be integrated. The food quantity and the delivery time can be individually programmed for each cage according to the scientific protocol: through a computed program, mice received during all the experimental period 5 grams of food each day. Once finished, the old food bars have to be replaced by new ones through six openings located in the MDS Front Panel (about every 15–20 days), in order to support the whole mission duration. During the first MDS mission the food bar composition was “Composition 4RF21” from Mucedola Srl, Settimo Milanese, Italy (cereals 66,5%, vegetable proteins 18,2%, forage 7,5%, animal proteins 3,5%, vitamin and mineral mixture 3,2%, fats from soya oil 0,4% and amino acids 0,1% - metabolizable energy 2668 kCal/kg) [25]. The Air Conditioning Subsystem generates a continuous airflow through the cages. Air speed is programmable in the range from 0.2 to 0.3 m/s and is used to flow fresh air through the cages. Generated CO_2_ is removed and consumed O_2_ is injected. The correct CO_2_ and O_2_ concentrations are maintained by exchanging with the ISS cabin about 5% of the total air circulating inside MDS every 2 minutes. Ejected and injected air flows are filtered by HEPA filters in order to prevent microbiological contamination from the ISS cabin to MDS and vice-versa. The ACS has also the important role to remove waste products (urine, feces, hairs, food debris etc.) from the cages. Waste products removed by the airflow are collected within Waste filters located below each cage. Exhausted Waste filters are exchanged every 30 days.

The ACS controls the temperature of the air flowing through the cages in the range from 25 to 30°C (lower value depends on ISS environment conditions). Passive control of relative humidity is performed by means of a desiccant integrated in the Waste filters. In standard ISS cabin conditions, the relative humidity is expected to stay in the range between 40% and 70%, the real value depending on the relative humidity of the air injected from the ISS cabin. The Illumination Subsystem implements light/dark cycles programmable in steps of 5 min starting from nominal 12 h light/12 h dark. Light intensity is programmable from 0 to 40 lux in steps of 10 lux. Diffuse light is provided during light periods (no bright spots). In addition, infrared light sources can be switched on for mice observation during dark periods. The Observation Subsystem permits the observation of mice during both, light and dark periods, by means of 6 video cameras (one camera/cage). Video data are acquired from 1 camera at a time and can be used to check mice health status and behavior as well as to check cage internal conditions and food presence. The duration and the start time of each observation session can be programmed according to the scientific protocol. Depending on the “visibility condition” of the ISS from a ground-station, acquired video data can be transmitted to ground in near real-time or stored on board an ISS mass memory for a subsequent transmission. The Payload Control Unit provides a high degree of autonomy and minimizes the need for crew intervention during experiment execution. PCU basic tasks are the execution of commands sent from ground or stored in the MDS internal memory, the acquisition and transmission to ground of telemetry data, the continuous monitoring of the experiment and facility status, the control and management of the environmental conditions important for mice well being (air speed and temperature, illumination levels, food delivery, control of main water tank status etc.) and the management of the interfaces with the ISS (mainly power and data) and with the crew. Biocompatibility of these subsystems were tested by hosting animals for various durations (14-days, 30-days, and 100-days) at three payload developmental stages starting from its breadboard, a ground prototype utilized to identify possible critical subsystems integrated in the MDS payload, [26] to the engineering model and the flight model [27].

### Flight Opportunity

The science that can be conducted using the MDS system can provide critical data for understanding physiological changes that occur in various tissues after a long duration spaceflight and contribute information that could be used to reduce health risk to crew members during future long duration space missions to the Moon and Mars. In particular, the possibility to conduct a near 100-days experiment with mice on board the ISS allowed to better improve our knowledge in the space biology field. In its first mission, MDS reached the ISS on board of Shuttle Discovery Flight 17A/STS-128 on August 28^th^, 2009. MDS landed on November 27^th^, 2009 with Shuttle Atlantis Flight ULF3/STS-129 after 91 days, thus performing the longest duration animal experiment in space.

### Animals Selection and Animal Experiment Ethical Approval

Pleiotrophin (PTN) is a heparin-binding cytokine [28] with different functions expressed by several types of cells in an early differentiation stage which is up-regulated in tissue injury and wound repair. Moreover, PTN is specifically involved in bone formation [29], neurite outgrowth [30] and angiogenesis [31] and stimulates proliferation and differentiation of human osteoprogenitor cells “in vitro” [32]. Transgenic mice selected for the MDS experiment over-express PTN under the control of the human bone specific osteocalcin promoter (PTN-Tg). Female PTN-transgenic mice present a faster bone formation process, while male mice show an increased bone mineral density. Furthermore, in ovariectomized transgenic mice the low estrogen concentration leads to a decreased bone tissue reduction compared to ovariectomized Wt mice [33]. As these transgenic mice show an increased bone mass and mineralization and are apparently more protected from experimentally induced osteoporosis, we select them to investigate whether genetic elements promoting bone formation can also protect the mice from space related osteoporosis. In particular we wanted to evaluate if PTN-Tg mice are differently susceptible to the negative effects of microgravity on bones compared to Wt mice and if the PTN over-expression could have a potential role in counteracting bone loss observed in microgravity. Not all mouse strains are equally suitable for flight experiments. Preliminary tests were performed utilizing a MDS breadboard in order to evaluate the behavior of different mouse strains. In contrast to mice of other strains, including the C57BlJ10 strain, the original PTN-transgenic mice developed by Hashimoto-Gotoh (Kyoto, Japan) in a BDF strain and the BDF Wt mice themselves did not tolerate well the MDS housing conditions. Considering that the demonstrated well-being of the animals housed in the MDS breadboard was one of the most important prerequisites to meet in order to be able to perform the MDS flight experiment, at the animal vivarium of National Cancer Research Institute (IST) in Genova, the original transgenic mice with the BDF genetic background were backcrossed in the C57BlJ10 strain to obtain a strain of PTN-Tg with a C57BlJ10 genetic background. Subsequent tests performed to validate the definitive MDS payload confirmed the suitability of the Wt and C57BLJ10/PTN (PTN-Tg) strains for the housing condition of the MDS hardware [29]. Twenty female and 10 male C57BLJ10/PTN transgenic mice were delivered to Charles River Laboratories (Wilmington, MA, U.S.A.) six months prior the forecasted Shuttle launch data. At the Charles River Laboratories (CRL) a colony was established from which the animals intended to be utilized for the flight experiment and to stay on the ISS were derived starting from embryos. Animal breeding and husbandry activities were performed with procedures preventing possible animal infection disease. Fifteen 5 weeks old transgenic and fifteen wild-type male mice of the same age were shipped from CRL to NASA-KSC Science Laboratories 3 weeks prior the forecasted launch date to allow mice to undergo one week adaptation to the new environment and about 2 week training before final animal selection for launch operations. Repeated bi-weekly shipping were planned in order to have mice with a correct age available also in case of Shuttle launch delays. Animals that did not participate to the flight experiment were delivered to the animal facility of IST. During the housing period at SLSL, mice were kept in rooms characterized by 20–24°C temperature, 40–60% relative humidity and 12 hours of light/dark cycle. Food (Mucedola srl, Milan, Italy) and water were provided ad libitum to all mice but the animals housed in the MDS modules during flight and ground control experiment. In these last cases only water was provided ad libitum while 5 g/die food was automatically provided by the MDS module. During the ground and vivarium pre-experimental period, mice were kept into IVC system cages of 300 mm×160 mm×140 mm while cages of 330 mm×150 mm×120 mm were used for the vivarium control experiment.

In all phases of the experiment (pre-flight, during the flight and post-flight) handling of animals was in accordance with the principles expressed in the “Guide for the care and the use of laboratory animals” (Office of Science and Health Reports of the USA National Institute of Health, Bethesda, USA). The approval of the MDS experiment was requested and obtained by the American Institutional Animal Care and Use Committee (IACUC) with protocol n°FLT-09-070(KSC) as well as by the Ethics Committee of the Animal Facility of the National Institute for Cancer Research (Genova, Italy) and by the Public Veterinary Health Department of the Italian Ministry of Health (protocol n° 4347–09/03/2009-DGSA.P.).

## Results

### Pre-flight Operations

Pre-launch operations were primarily done at the Kennedy Space Center (KSC) in the Space Life Science Laboratory (SLSL). Hardware final assembly and testing was done at the Space Station Processing Facility (SSPF). Pre-launch activities were performed by a composite team formed by staff from the ASI MDS Program Office, the Principal Investigator Team of the Genova University, and the Payload Developer Team of Thales Alenia Space. Activities started at the SSPF on August 4^th^, 2009, with the inspections and the functional tests of both flight and the spare MDS models (FM and FS). Following the above check, aimed to verify that no damage had occurred during the shipment to KSC, the cooling water circuit was filled and tested for certification purpose. Further certification tasks, consisting of mass measurement and the execution of the leak test, were performed on the FS on August 10^th^ and 11^th^. The following day all the hardware was moved to the SLSL to get started with the mission launch preparation tasks. At the SLSL, both models were integrated with their respective ground support equipment: mechanical (MGSE), fluidic (FGSE) and electrical (EGSE) (Figure S2).

The first scheduled launch date was August 26^th^. After their arrival at the KSC-SLSL 3 weeks prior that date, the mice were housed in special training cages reproducing in size and for food and water delivery systems the Mice Chamber habitat of the MDS payload (Figure S3). Food and water consumption was daily monitored in order to make sure that animals learned how and where to find food and water. After the training period, 6 Wt and 6 PTN-Tg mice were selected for insertion in the MDS Flight Model (FM) and Flight Spare (FS) model. Selected mice were those that during the training gained more weight and drank more water. Three Wt and three PTN-Tg mice were inserted in each model. During their housing in the FM and FS models mice were daily weighted and their food and water consumption checked. The two flight models, already containing the animals, underwent final technical check of all the subsystems and the Daily Operating Table (DOT - containing the working parameters for the food delivery system, the dark-light cycle of the illumination subsystem and other instruction relevant to the facility automatic functions) was reviewed. Eventually the models were uploaded on the MDS Payload Control Unit (PCU). On August, 23^rd^, in preparation of the launch, in the primary flight model FM the waste filters were replaced, the potable water system filled with 250 ml of water and one loose food bar per cage inserted. The FM model was then installed in its carrier, turned over to NASA personnel and transported to the launch pad 39A per installation on the assigned locker into the Discovery orbiter middeck. Following the announcement of a 72-hour launch scrub, due to a false indication from a fuel tank sensor of the STS-128, the flight model was returned to the MDS team. While the FM model was being refurbished, the launch preparation procedure was repeated with the spare flight FS model. On August 26^th^, the FS model carrying the other six mice was turned-over to NASA for the second launch attempt scheduled on August 28^th^. The STS-128 launched at 11.58 p.m. of August 28^th^, 2009, transporting to the International Space Station the MDS facility and its living payload. A detailed description of all the principal pre-launch activities at SLSL is reported in Table 1.

**Table 1:** Pre-flight operations.

Day to launch	Activities (animal training and mds payload set up for launch)
−24	Arriving of 15 Wt and 15 PTN-Tg mice at SLSL and transfer of the animals to isolator cages.
−22	Transfer of 7 Wt and 7 PTN-Tg mice to the training cages.
−11	Transfer of 6 Wt and and 6 PTN-Tg trained to the Flight (FM) and the Spare (FS) MDS modules (3 Wt and 3 Tg to each module).
−11/−6	Daily check of the models subsystems and of the health status of animals inside the FM and FS MDS modules.
−5	Replacement of the waste filter. Filling of the water system. Insertion of food bars. Transport of FM MDS to the launch pad to be installed on the Discovery orbiter middeck. Check of the health status of animals inside the FM and FS MDS modules.
−4	Return of the FM MDS module to SLSL due to a 72 hours scrub. Removal of the mice from FM MDS, refurbishment of the FM module as new spare payload and reinsertion of the animals.
−2	Transport of original FS MDS module to the launch pad to be installed on the Discovery orbiter middeck.
0	Launch of Shuttle Discovery with STS-128 crew on August 28^th^, 2009 at 11.58 p.m. (EDT)

Activities begun on August 4^th^, 2009. Acronyms reported in Table 1 are explained in Table S3.

### On-orbit Operations and Ground Support

During its travel to the ISS on-board the STS-128, the MDS was powered and operating on “survival*”* mode, meaning that only the minimal functions required for mice survival and wellness were activated. Therefore, since the observation subsystem was not operating, visual checks to verify the mice status were daily performed by the STS-128 crew. Since the food delivery system was one of the MDS functions that were suspended during the “survival” mode period, to ensure food availability to the mice, a food bar was inserted in each cage. Four days after launch, following the docking of the Discovery to the ISS, MDS was transferred from the Shuttle Middeck to the Express Rack 4 in the Japanese Experiment Model (JEM). The operating mode of the system was then switched to *“*experiment-autonomous*”* mode, thus activating all system functions. Because of the automated operations of the MDS model, only a minimum intervention of the astronauts on the payload itself was required (Figure S4). In fact, based on the preliminary tests conducted on ground with the engineered MDS model, the operation plan included only few nominal maintenance operations to be executed by the crew: the potable water refill scheduled to take place every 9 days, the food cartridges replacement every 19 days and the waste filters replacement every 30 days [27]. Other procedures, considered on-need maintenance operations, for which astronauts underwent a specific pre-flight training, were the removal from the cage of an expired mouse, the video camera cleaning, the food bar manual advancement and the visual check of mice and cage internal status. The on-orbit operations were constantly supported by personnel at User Support Operations Center (USOC) in Naples, User Home Base (UHB) in Genova and Payload Support Center (PSC) in Milan and coordinated by ASI. Three video observation sessions (2-hour each) per day were recorded and downloaded permitting the MDS ground team to check frequently mice well-being and cages internal status. Thanks to this function, we could save one of the mice whose paw was trapped against the roof of the cage by the food bar. In this case, the observation made by the ground that a mouse was blocked in a corner of its cage and the immediate intervention of the astronaut were of primary importance.

Unfortunately, during the 3 months period on the ISS, one PTN-Tg (PTN-Tg3 at day 24) and two Wt mice (Wt3 at day 16 and Wt1 at day 44) died. The three dead mice were removed from the cage by the astronauts and cryo-conserved by placing them in a -20°C refrigerator. After the MDS landing, PI and NASA veterinarians performed a necropsy of the dead mice. The necropsy revealed that mouse Wt3 had a major spinal cord lesion possibly occurred during the shuttle lift off. Due to the difficulty of making significant observations on dead mice, which were frozen and thawed, nothing significant could be noticed in the case of PTN-Tg3 and Wt1 mice. Nonetheless, a subsequent analysis of feces present in the cage of the Wt2 mouse suggested that the animal could have developed a liver pathology. The post–landing checking performed on the mice cages revealed that Wt1 died in consequence of a failure of the food cassette system. In fact, whereas the water dispenser and the FDS were operating nominally in all other cages, a failure of a Teflon washer, preventing the automatic movement of the food bar, was observed in the Food Envelope (FEV) of cage 3. The remaining three mice showed a normal behavior throughout the all mission and appeared in excellent health conditions at landing.

Water consumption was daily checked and recorded (Figure S5). Monitoring of water consumption was critical for the control of the animal well being in flight, to confirm animal death and to notice and solve the problem with the animal whose paw was blocked between the food bar and the cage roof. A short chronicle and a description of the major events occurred during the on-orbit operations are reported in Table S1.

### Animal Behavior During the on Orbit Phase of the Mission

The behavior repertoire of wild type and transgenic mice housed in the MDS has been video-captured in-flight with the MDS Observation Sub System, 6 video cameras monitoring animals in each chamber, in real time. Image sequences (sent from the ISS to Telespazio SpA in Naples, Italy) were downloaded and converted to video (.avi format) by using a free-software (VideoMatch vs 5.5.3, SICS, Italy). The behavior patterns characterizing mice in the MDS system were analyzed during the initial, middle and late phase of the 91-day experiment. An average of 10,500 jpg images a day (acquisition rate 10 frames/second) for each animal taken between 00:00–02:00 a.m., 08:00–10:00 a.m., 04:00–06:00 p.m. were converted in video (Video S1) of about 1 min each and carefully scored by a highly trained observer using a dedicated ethological software (“The Observer”, Noldus, Netherlands). While vertical activity such as “rearing” or “wall rearing” (standing on hind legs and placing forelimbs on the wall of the cage) as well as “digging”, were obviously virtually suppressed, frequency and duration of behavior of the following (fully characterized on ground) behaviors, were observed and recorded: “exploring”: moving around the cage; “self-grooming”: wiping, licking, combing any part of the body; “face washing”: self-explanatory; “sniffing”: sniffing the environment; “resting”*:* no visible movements (either closed or open eyes); “eating and drinking”*:* self explanatory. Moreover, several behaviors, characteristic of the microgravity environment were observed and defined: “floating”: swinging in the air, without holding the cage and no visible movement*;* “hanging”: holding the bars of the metal top of the cage with the forelegs, stopping in this position (body suspended); “bar-walking”: moving holding the bars of the grid of the cage with the fore- and hind-paws; “on cookie”: grasping, surfing, staying, eating the cookie, when present. Animals learned quickly to manage with microgravity environment using the grid to grasp and/or direct their movements and appeared to fit well in space. As long as the cookie was present in the cage, they clearly showed a preference to interact with it, suggesting the need to develop some *ad hoc* object to insert in the cage in future missions. All mice spent most of the time in the proximity of where water and food were present in the cage. Behavior repertoire was largely conserved in space with the emergence of “floating” and frequent rolling movements. Together with preliminary ground-based data, differences in behavior profile between Wt and PTN-Tg mice were observed. Most notably PTN-Tg mice were apparently more active than wild-type controls on ground. In space, although the small number of animals makes impossible to carry out statistical analysis for behavior parameters, it appeared that PTN-Tg mice consistently kept floating more than Wt ones especially in the last period of the experiment (Figure S6 B). While in water, rodent floating behavior is generally associate with passive behavior, anhedonia and stress response, in space it could reflect environment adaptation, being also a strategy to direct movement with reduction in energy effort. Thus, the increased floating behavior in PTN-Tg mice could indicate a different coping strategy to adapt to an altered gravitational environment. On the other hand, grooming behavior, which is a classic index of displacement activity, appeared more pronounced in wild type animals again in the last period of the experiment. It is of notice that the *ex post* detailed observation of the behavior profile in the days preceding the death of the two Wt mice and of the PTN-Tg mouse did not evidenced any warning sign.

### Post-landing Activities and Tissue Sharing Program Implementation

**Figure 2:**
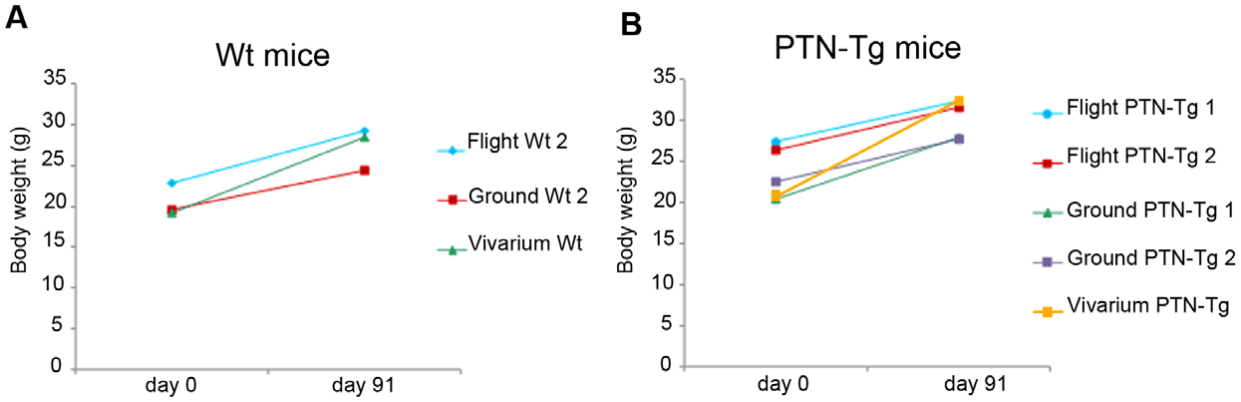
Body weight. Body weight increase of the flight mice and of the ground and vivarium control mice during the 91-day MDS experiment. Panel A): Wt mice; Panel B): PTN-Tg mice.

On November 27^th^, Shuttle Atlantis and the STS-129 crew landed at KSC bringing back to Earth the MDS payload containing the mice. The MDS model was handed over by NASA to the MDS team and was available at the SLSL within 150 minutes from landing. After the arrival of the payload at the SLSL, mice were extracted from the cage and the post-landing science operations started. Video images of the mice immediately after extraction from the MDS payload clearly show the difficulty of the mice in moving once returned under Earth gravity condition (Video S2). The TSP operations began with the immediate collection of the urine of the three live mice and the measurement of their weight. In Figure 2 the pre- and post-flight body weight of each mouse is shown and compared with the body weight increase of the mice from the ground and the vivarium controls (see following paragraph). It is to note the slightly reduced gain of weight observed in all mice housed in the MDS payloads compared to animals maintained in standard vivarium cages. Apparently no specific effects on the animal weight due to the near-zero gravity were observed. The sequence of the subsequent mice processing was: PTN-Tg1, Wt2 and PTN-Tg2. The whole processing of each mouse lasted about 90 minutes. The veterinarian drew blood from the orbital sinus and mice were then euthanized by CO_2_. The following tissues were dissected and processed according to each TSP-PI instructions: bone, bone marrow, brain, calvaria, claw, diaphragm, heart, hypophysis, intestine, kidney, liver, lung, muscle adductor longus, muscle forelimb, muscle hindlimb, pancreas, forelimb, skin, spleen, stomach, adrenal glands, testis, thymus, trachea, thyroid. After the first three mice tissue dissection, the dissection activity continued by collecting the bone samples from the three mice that died on orbit: Wt1, PTN-Tg3, Wt3. All samples (more the 350 samples in 30 boxes to be maintained at different temperatures) were shipped for subsequent analysis from the SLSL to the 20 PIs in 13 cities of 6 different countries (Figure S7). Additional information on the distribution of the samples to the different research groups is reported in Table S2. All acronyms reported are explained in Table S3.

### Ground and Vivarium 1 g Control Experiments

A 1 g ground replica of the flight experiment (“ground control”) was performed in the FM MDS model at the University of Genova from November 13^rd^, 2009 to February 11^st^, 2010. As additional control, tissue samples were collected also from mice maintained in standard vivarium Individual Ventilated Cages (IVC) (“vivarium control”). The two different control experiments were performed in order to distinguish between a possible MDS housing effect on the mice and the effect of the microgravity exposure. The animals for the ground control were chosen on the basis of their capacity of adaptation to the habitat. This capacity was tested by placing the mice in the same training cages used for the flight mice training. The ground control experiment was designed to repeat exactly everything occurred to mice during the on-orbit experiment. Therefore, two wild type and one transgenic mice, were sacrificed at the same experimental day than the corresponding flight mice death and placed in a -20°C refrigerator. As for the flight experiment, all MDS habitat parameters, including water and food consumption, as well as images derived from the camera observation system, were registered by the computer connected to the FM MDS model. The vivarium control experiment was conducted in parallel to the ground control. In this case three wild type and three PTN transgenic mice were maintained in standard vivarium conditions for the all 91 experimental days. Water and food consumption was daily checked and data were collected to be compared to the values derived from the flight and the ground experiments. The weight increase of all ground and vivarium experiment animals after the 91-days period was determined. At the end of the 91-day experiment the TSP procedures already performed on the flight animals was repeated on the ground and vivarium controls animals.

## Discussion

The Mice Drawer System project is an ASI funded program that was established to support long-duration scientific experiments with mice on board the International Space Station. Studies conducted in microgravity conditions using mice as a model may advance our knowledge on changes occurring in various physiological systems after exposure to a near-zero gravity environment. Eventually this could also lead to a reduction of the health risk to crewmembers during long duration space flights. The first MDS mission lasted 91 days, marking it the longest duration animal experiment in space. Observations made by analyzing the effect of the microgravity exposure on different organs and tissues are the subjects of some independent accompanying papers. Undoubtedly, the limited number of mice (three wild type and three PTN-tg mice) that the MDS payload could house during the flight, together with the fact that unfortunately only three mice (one wild type and two PTN-Tg mice) survived to the 91-day spaceflight, represents a critical aspect of the experiment, especially for the statistical reliability of the collected data. However the MDS experiment was a unique opportunity to study the microgravity long-term exposure effects on several tissues of an animal model and to collect interesting observations that could prepare the field to future experiments. Hopefully future re-flight of MDS will be possible to increase the number of analyzed animals thus increasing the significance of the made observations.

## Supporting Information

Figure S1 **MDS cage interior.** Interior of a cage for individual mice housing in the MDS model. Details of the water delivery and of the food delivery systems are shown.
(TIF)

Figure S2 **MDS models.** MDS FM and FS models installed on Mechanical Ground Support Equipment with reduced Fluidic Ground System Equipment mounted on the top. The MDS models contain already the mice and are ready to be installed in their carrier and turned over to NASA personnel.
(TIF)

Figure S3 **Training cage.** Mouse inside a training cage for adaptation to the new environment and training on water and food delivery systems.
(TIF)

Figure S4 **MDS on International Space Station.** Astronauts Nicole Stott and Bob Thirsk checking the MDS payload installed into Express Rack 4 in the Japanese model (JEM) on board the ISS four days after the launch of Shuttle Discovery (courtesy by NASA).
(TIF)

Figure S5 **Water consumption data.** Water consumption of mice inside the MDS flight model during their permanence in the ISS.
(TIF)

Figure S6 **Mice behavior during flight period.** Mice behavior during the experimental period. A) eating activity duration, B) floating activity duration C) grooming activity duration.
(TIF)

Figure S7 **Tissue Sharing Program participants.** Tissue Sharing Program team at KSC-SLSL.
(TIF)

Table S1 **On-orbit activities.** Activities on the ISS began on September 1^st^, 2009, four days after the launch of the Shuttle Discovery.
(DOC)

Table S2 **Tissue Sharing Program (TSP)**. Tissue Sharing Program (TSP) involved investigators and tissues they are interested in.
(DOC)

Table S3 **Acronyms.** Acronyms reported and their meaning.
(DOC)

Video S1 **Mouse behavior during flight experiment daily observation.** Mouse behavior inside MDS cage during flight daily observation.
(AVI)

Video S2 **Mouse behavior upon landing.** Mouse Wt2 behavior immediately after extraction from MDS payload upon landing.
(AVI)
