# Supplementary figures and images for: The Mice Drawer System (MDS) Experiment and the Space Endurance Record-Breaking Mice
Source: PLoS One. 2012 May 29;7(5):e32243. doi: 10.1371/journal.pone.0032243 (PMC3362598; doi:10.1371/journal.pone.0032243)

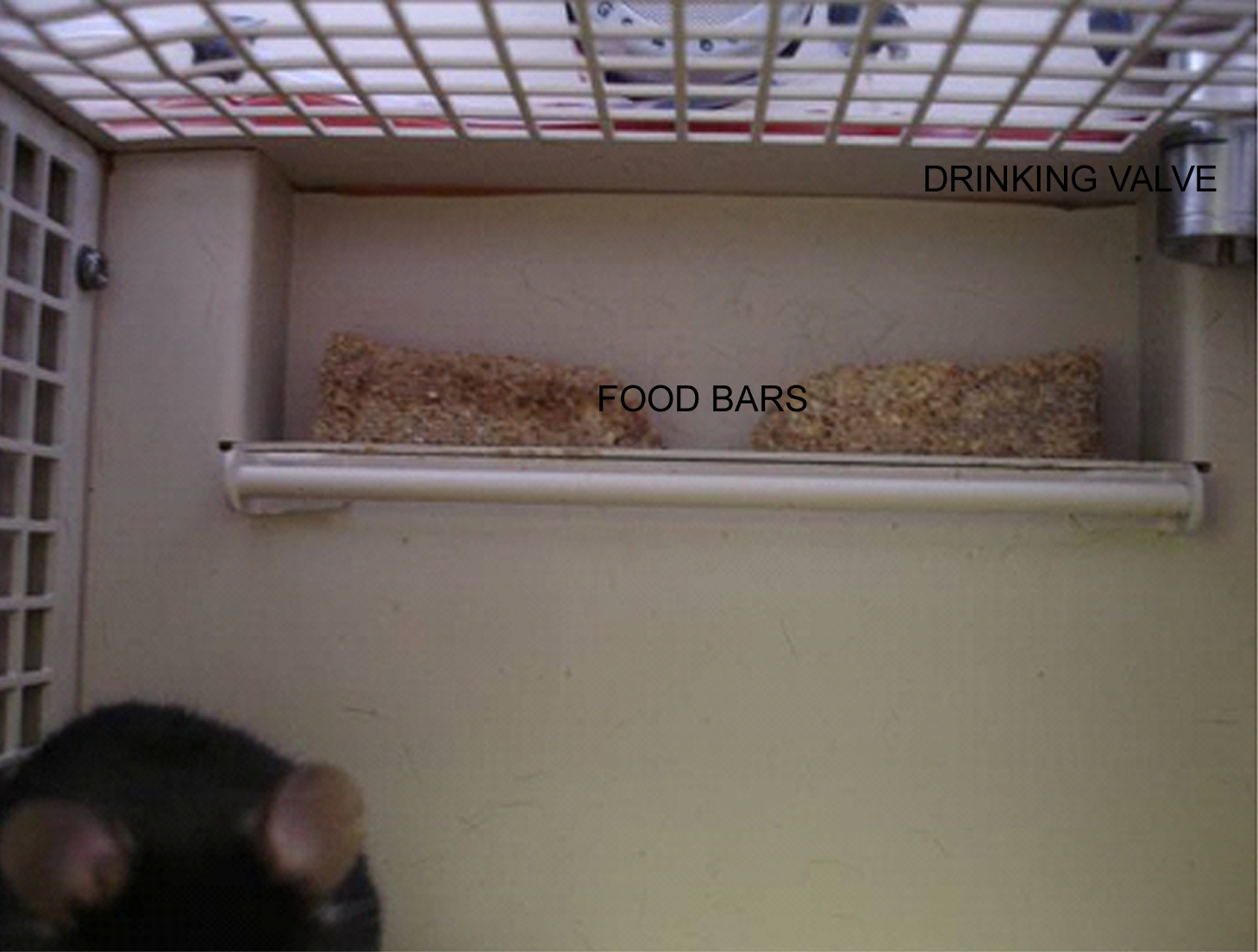

Supplement: Figure S1 — MDS cage interior. Interior of a cage for individual mice housing in the MDS model. Details of the water delivery and of the food delivery systems are shown. (TIF) [file pone.0032243.s001.tif]

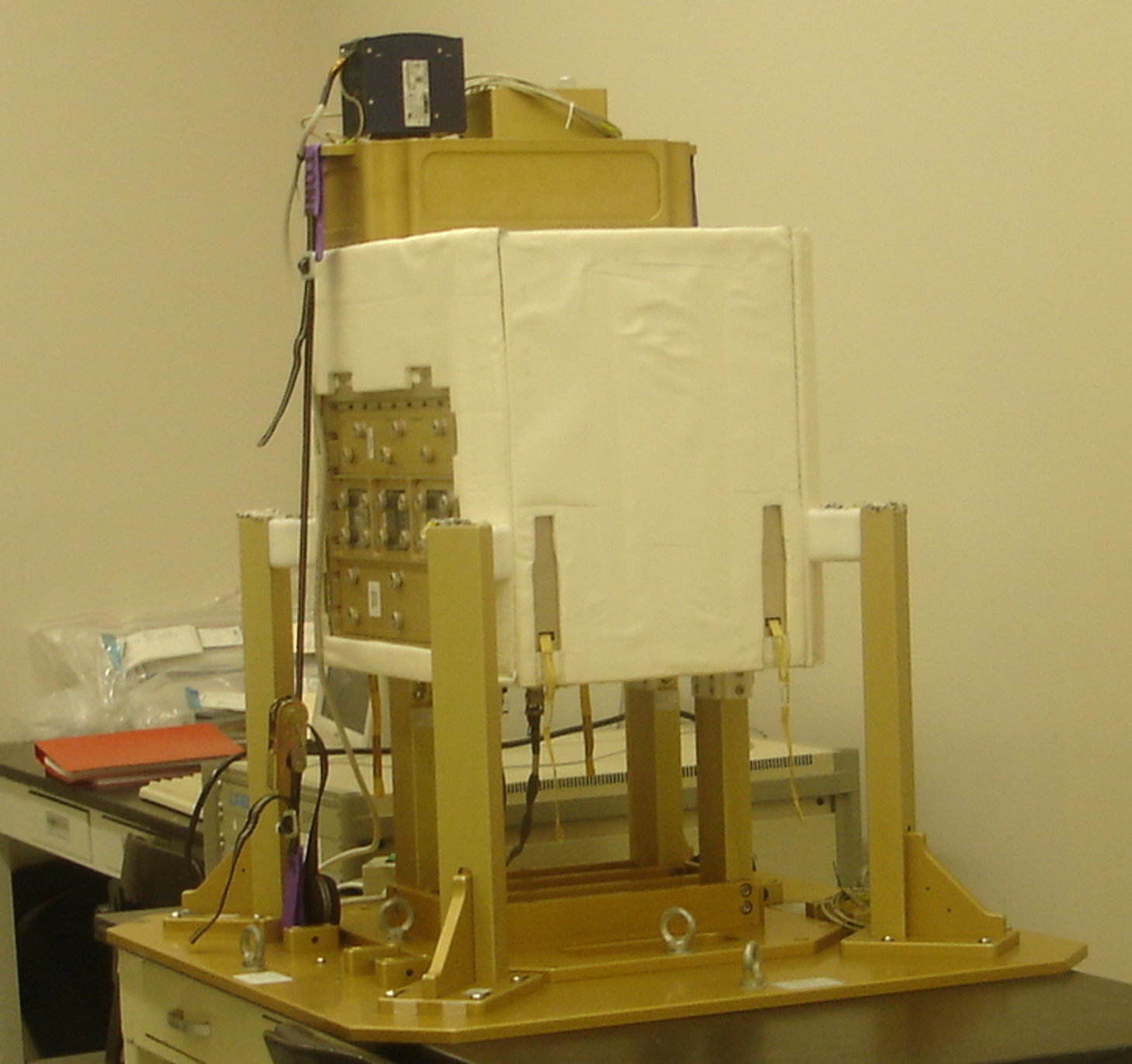

Supplement: Figure S2 — MDS models. MDS FM and FS models installed on Mechanical Ground Support Equipment with reduced Fluidic Ground System Equipment mounted on the top. The MDS models contain already the mice and are ready to be installed in their carrier and turned over to NASA personnel. (TIF) [file pone.0032243.s002.tif]

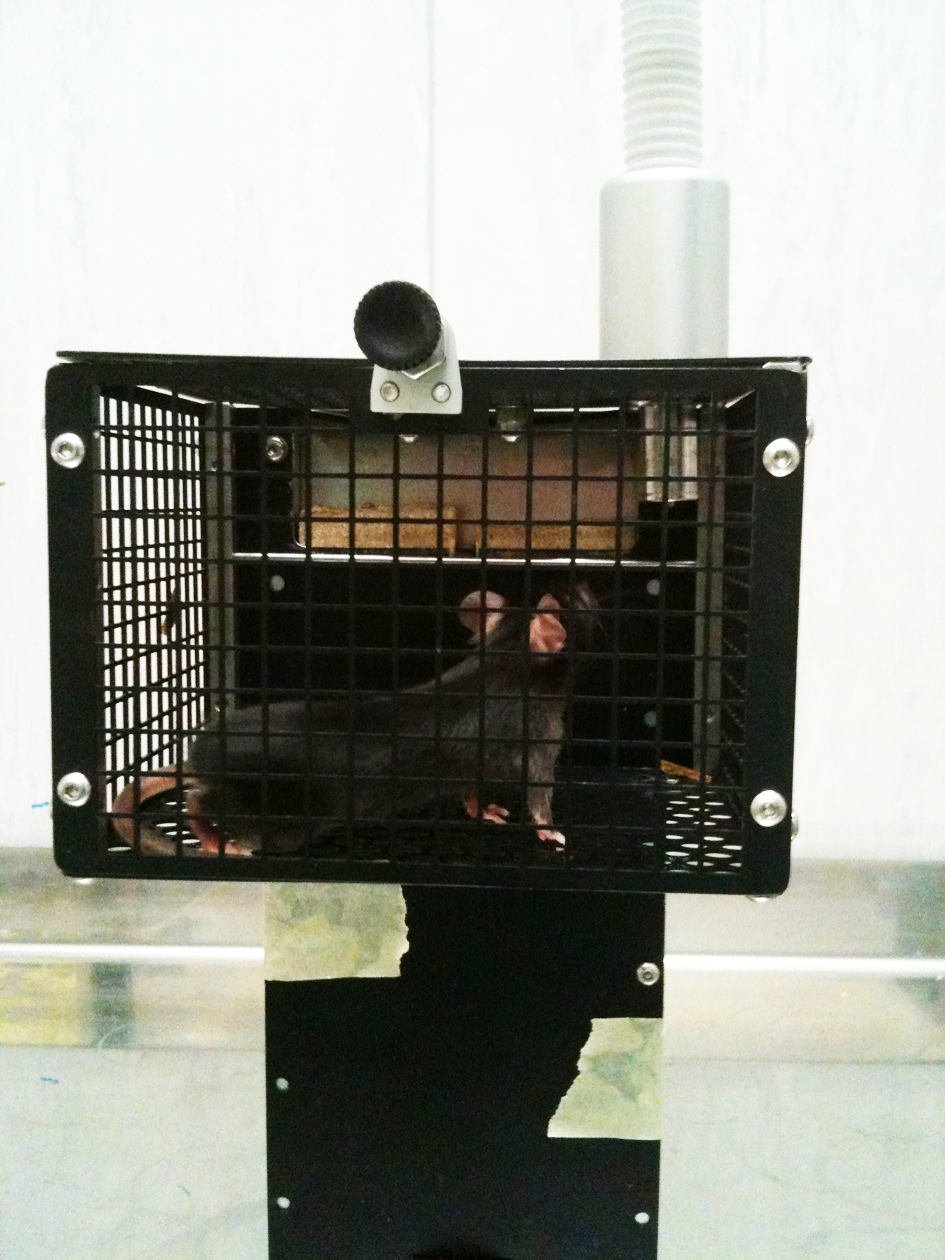

Supplement: Figure S3 — Training cage. Mouse inside a training cage for adaptation to the new environment and training on water and food delivery systems. (TIF) [file pone.0032243.s003.tif]

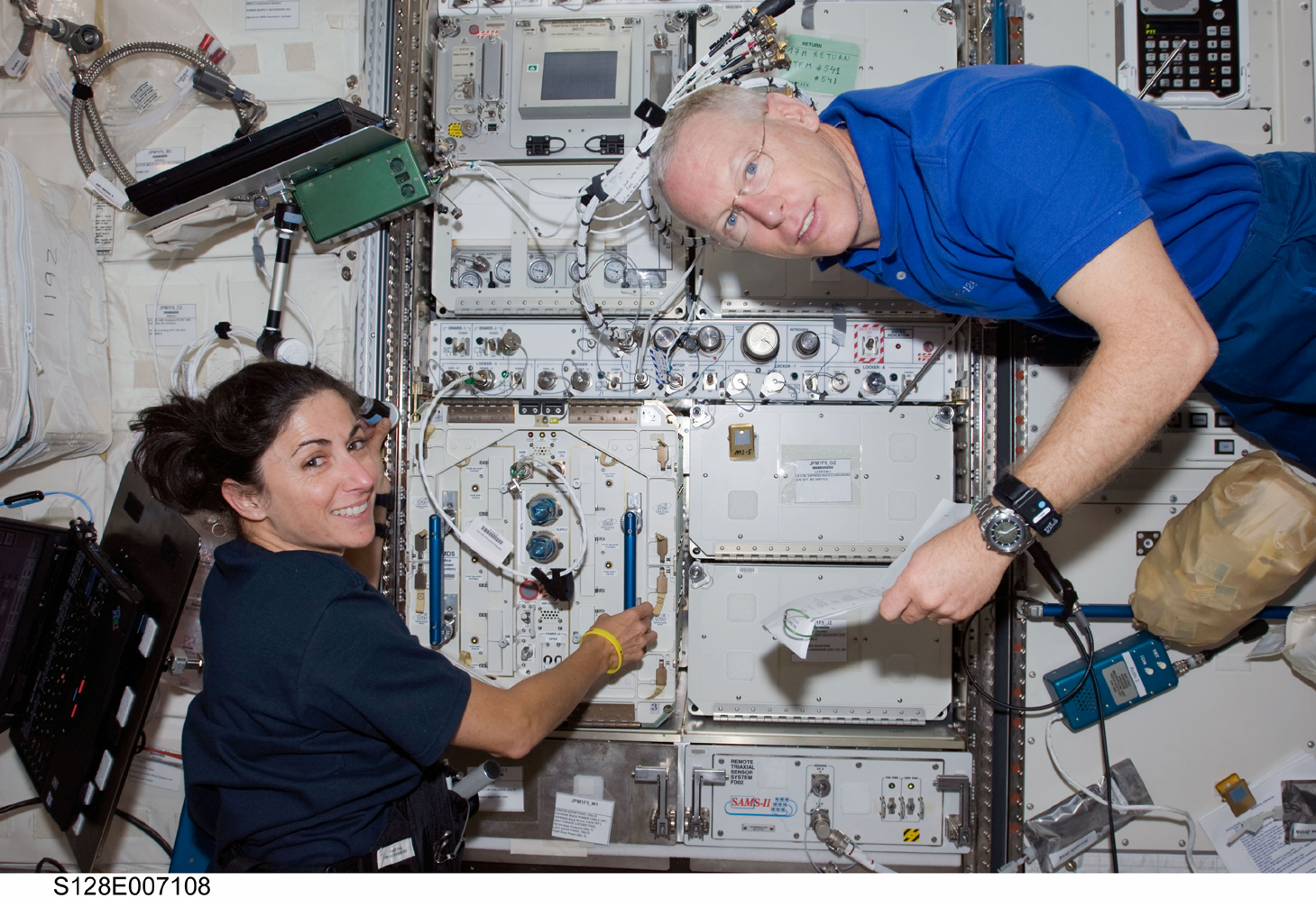

Supplement: Figure S4 — MDS on International Space Station. Astronauts Nicole Stott and Bob Thirsk checking the MDS payload installed into Express Rack 4 in the Japanese model (JEM) on board the ISS four days after the launch of Shuttle Discovery (courtesy by NASA). (TIF) [file pone.0032243.s004.tif]

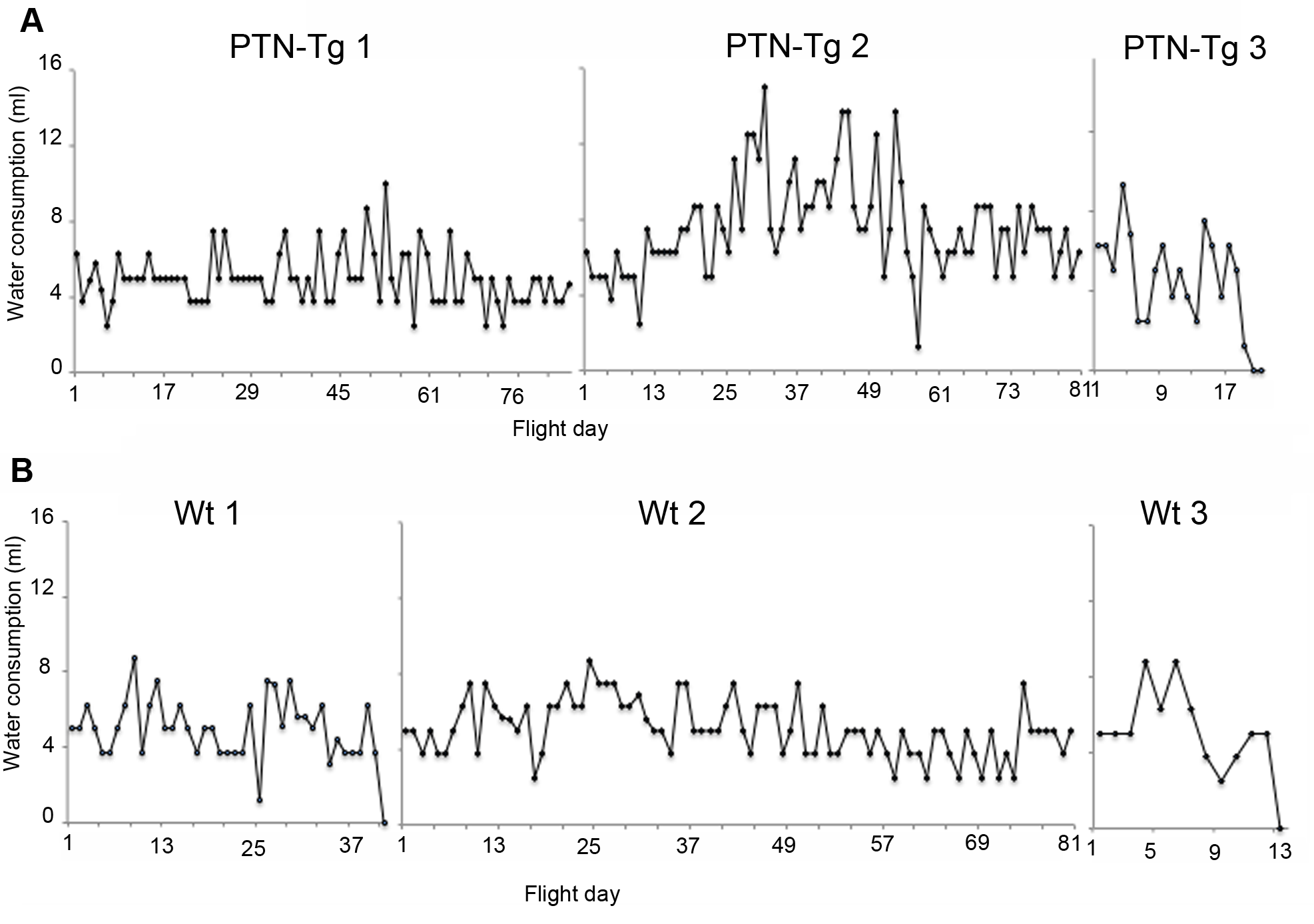

Supplement: Figure S5 — Water consumption data. Water consumption of mice inside the MDS flight model during their permanence in the ISS. (TIF) [file pone.0032243.s005.tif]

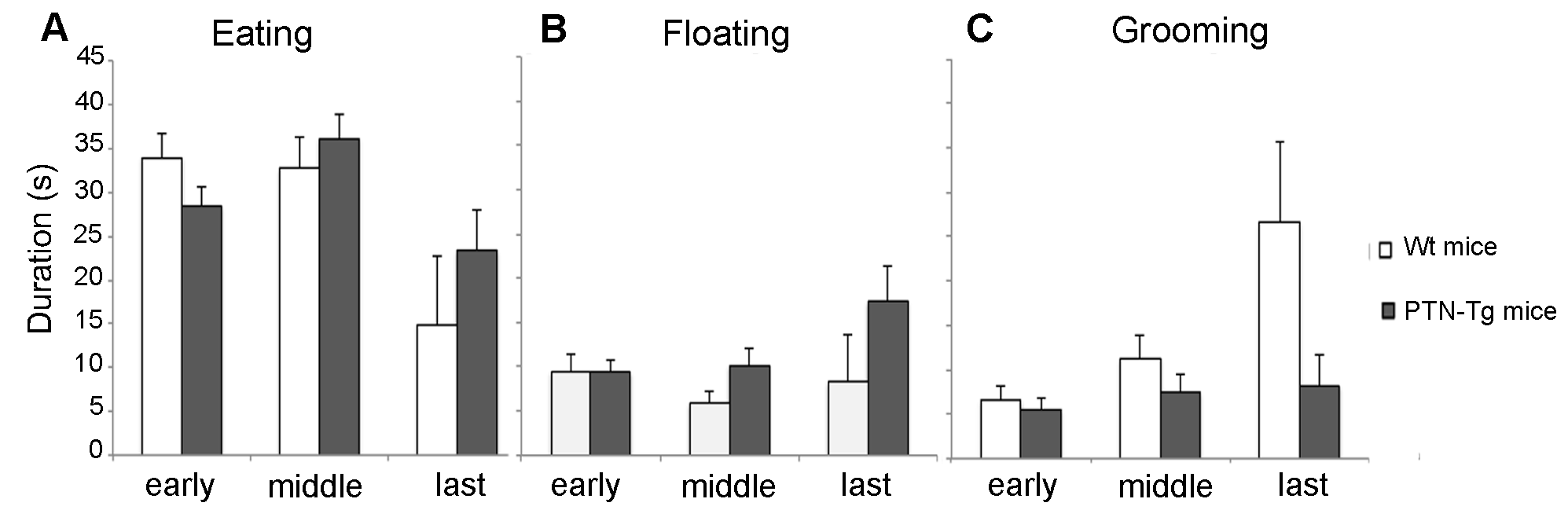

Supplement: Figure S6 — Mice behavior during flight period. Mice behavior during the experimental period. A) eating activity duration, B) floating activity duration C) grooming activity duration. (TIF) [file pone.0032243.s006.tif]

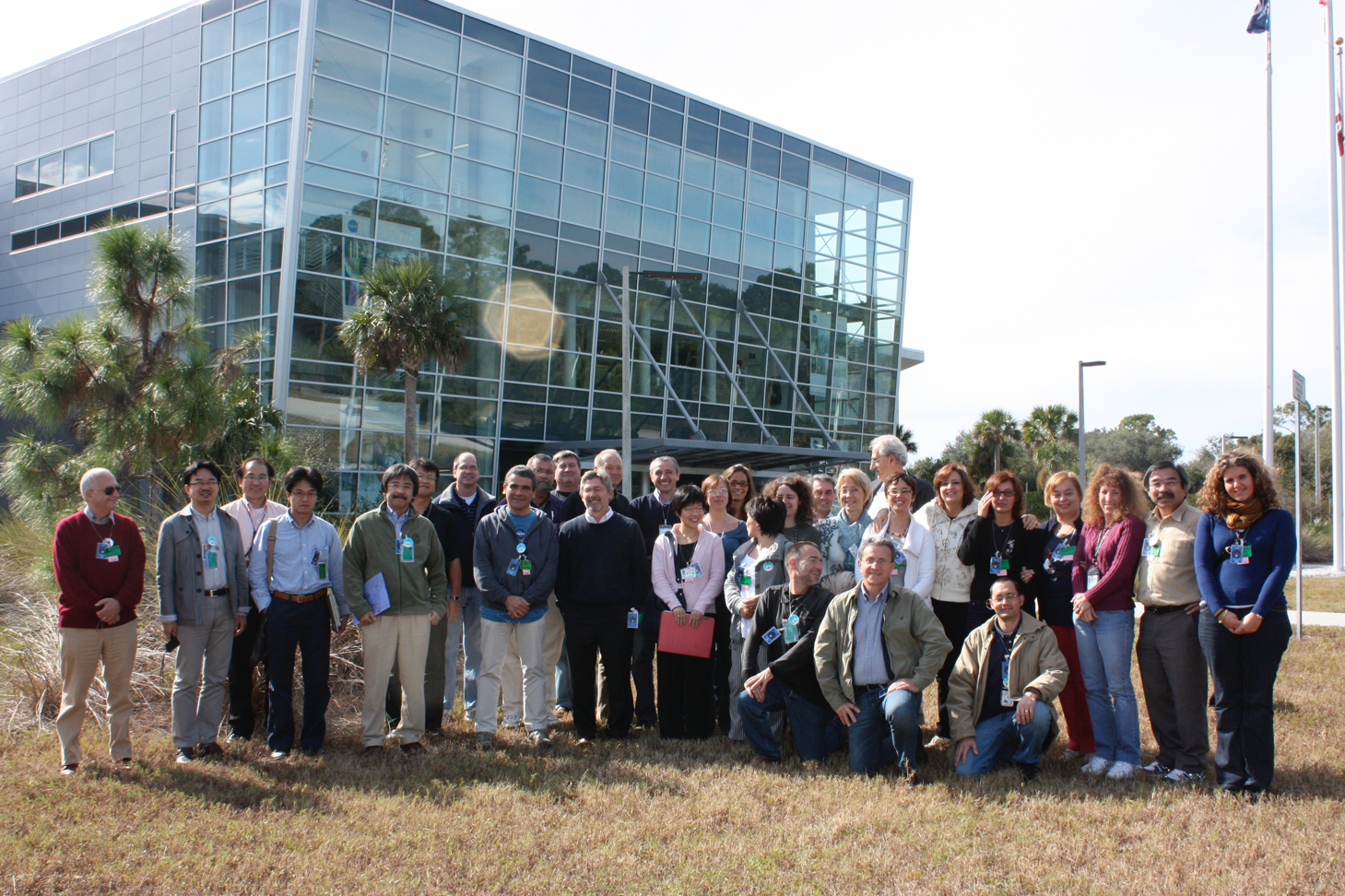

Supplement: Figure S7 — Tissue Sharing Program participants. Tissue Sharing Program team at KSC-SLSL. (TIF) [file pone.0032243.s007.tif]
