# Supplementary material for: The Mice Drawer System (MDS) Experiment and the Space Endurance Record-Breaking Mice
Source: PLoS One. 2012 May 29;7(5):e32243. doi: 10.1371/journal.pone.0032243 (PMC3362598; doi:10.1371/journal.pone.0032243)
Supplement: Table S1 — On-orbit activities. Activities on the ISS began on September 1st, 2009, four days after the launch of the Shuttle Discovery. (DOC) [file pone.0032243.s008.doc]

**Table S1:** **On-orbit activities.** Activities on the ISS began on September 1^st^, 2009, four days after the launch of the Shuttle Discovery.

| **Flight day** | **Flight activities** |
| --- | --- |
| 4 | Transfer of the MDS model from the Shuttle to the Japanese Experiment Module (JEM) of the ISS. Reconfiguration of the hardware to “Experiment” mode. Insertion of six Food Envelopes (FEVs) in the Food Delivery System (FDS). Filling of the Potable Water System (PWS). Replacement of the FEV in cage #3 due to a malfunctioning of the first FEV inserted. Connection of the cooling system to the express rack. |
| 7 | Retraction of food bars after a visual check because of an only partial consumption of the loose food bars placed in the cage before launch. |
| 9 | Advancement of food bars to expose them back to the mice. |
| 11 | Manual advancement of food bar in cage #3. |
| 16 | Death of Wt 3 mouse in cage #6. Removal of the animal from the MDS model performed by crew. |
| 20 | Replacement of exhausted FEVs. Insertion of new FEVs. Advancement of food bars. |
| 24 | Death of Tg 3 mouse in cage #3. Removal of the animal from the MDS model performed by crew. |
| 25 | Replacement of the exhausted waste left side filter. Reconfiguration of MDS in nominal configuration. |
| 27 | Replacement of the exhausted waste right side filter. Reconfiguration of MDS in nominal configuration. |
| 29 | Check of the water delivery system on cage #4. |
| 30 | Check of the air fan system due to high values of relative humidity and C0_2_ concentration. |
| 32 | Check of the air fan system due to high values of relative humidity and C0_2_ concentration |
| 35 | Replacement of exhausted FEVs. Insertion of new FEVs. Advancement of food bars. Cleaning procedure for MDS camera lens and illumination subsystem LEDs. |
| 44 | Death of Wt 1 mouse in cage #4. Removal of the animal from the MDS model performed by crew. Checking of food bar exposure by cage inspection. |
| 48 | Replacement of exhausted FEVs. Insertion of new FEVs. Advancement of food bars. |
| 49 | A visual inspection performed by the ground team through the internal camera indicated trapping of mouse in cage #2 by the paw. A visual inspection of the animals by the crew confirmed the finding. Crew intervention to get the mouse free and to advance the food bar. |
| 53 | Visual inspection of the animals and manual advancement of the food bars performed by crew. |
| 54 | Replacement of the waste filters and refill of the PWS performed by crew. |
| 56 | Visual inspection of the animals and manual advancement of the food bars performed by crew. |
| 59 | Manual advancement of the food bars performed by crew. |
| 60 | Check of potable water valve of cage #2 performed by crew. |
| 61 | Removal of the front panel air outlet muffler to enhance acoustic performance, improve the air circulation and reduce internal built-in humidity. |
| 62 | Replacement of exhausted FEVs. Insertion of new FEVs. Advancement of food bars. |
| 63 | Refill of the PWS performed by crew. |
| 66 | Cleaning of the camera lens and LEDs windows. |
| 67 | Visual inspection of the animals performed by crew. |
| 70 | Visual inspection of the animals performed by crew. |
| 73 | Refill of the PWS performed by crew. |
| 74 | Replacement of exhausted FEVs. Insertion of new FEVs. Advancement of food bars. |
| 77 | Visual inspection of the animals performed by crew. Manual and remote controlled advancement of food bars. |
| 81 | Visual inspection of the animals performed by crew. Manual and remote controlled advancement of food bars. |
| 88 | Reconfiguration of MDS payload to “Descent configuration” and transfer from the Japanese Experiment Module (JEM) to the Atlantis orbiter middeck (STS-129). Switch of MDS model to the “Survival” mode. |
| 89 | Visual check of the animals performed by the STS-129 crew. |
| 90 | Visual check of the animals performed by the STS-129 crew. |
| 91 | Landing of Shuttle Atlantis on November 27^th^, 2009 at 9.44 am. |

Acronyms reported in Table S6 are explained in Table S12
