# Supplementary material for: The Mice Drawer System (MDS) Experiment and the Space Endurance Record-Breaking Mice
Source: PLoS One. 2012 May 29;7(5):e32243. doi: 10.1371/journal.pone.0032243 (PMC3362598; doi:10.1371/journal.pone.0032243)
Supplement: Table S2 — Tissue Sharing Program (TSP). Tissue Sharing Program (TSP) involved investigators and tissues they are interested in. (DOC) [file pone.0032243.s009.doc]

| **Principal Investigator** | **Country** | **Tissue** |
| --- | --- | --- |
| Ambesi | Italy | Thyroid |
| Cancedda | Italy | Serum, Bones, Bone marrow |
| Capasso | Italy | Urine, Kidney |
| Dinardo | Italy | Heart, Thoracic diaphragm |
| Miserocchi | Italy | Lung, Trachea |
| Pippia | Italy | Intestine, Stomach |
| Rizzo | Italy | Serum, Red blood cells |
| Santucci | Italy | Tongue, Brain, Surrenal glands, Hind paw |
| Schiaffino / Conte Camerino | Italy | Tongue, Limb muscles |
| Strollo / Masini | Italy | Pituitary gland, Pancreas, Kidney, Liver, Testis, Stomach, Forepaw |
| Bateman | U.S.A. | Bones |
| Blottner | Germany | Tongue, Limb muscles |
| Boyle / Shin-ici | U.S.A. | Skull |
| Green Johnson | Canada | Lymph nodes, Colon |
| Nusgens | Belgium | Skin |
| Ohira | Japan | Brain, Testis, Spinal chord, Limb muscles |
| Yufang | U.S.A. | Thymus, Spleen |

**Table S2:** **Tissue Sharing Program (TSP)**. Tissue Sharing Program (TSP) involved investigators and tissues they are interested in.
