# Supplementary material for: The Mice Drawer System (MDS) Experiment and the Space Endurance Record-Breaking Mice
Source: PLoS One. 2012 May 29;7(5):e32243. doi: 10.1371/journal.pone.0032243 (PMC3362598; doi:10.1371/journal.pone.0032243)
Supplement: Table S3 — Acronyms. Acronyms reported and their meaning. (DOC) [file pone.0032243.s010.doc]

**Table S3: Acronyms.** Acronyms reported and their meaning.

| **Acronym** | **Acronym meaning** |
| --- | --- |
| ACS | Air Conditioning Subsystem |
| ASI | Italian Space Agency |
| CRL | Charles River Laboratories |
| CSA | Canadian Space Agency |
| DOT | Daily Operating Table |
| EDT | [Eastern Daylight Time](http://wwp.greenwichmeantime.com/time-zone/usa/eastern-time/eastern-daylight-time.htm) |
| EGSE | Electrical Ground Support Equipment |
| ESA | European Space Agency |
| FDS | Food Delivery Subsystem |
| FEV | Food Envelope |
| FGSE | Fluidic Ground Support Equipment |
| FM | Flight Model |
| FS | Flight Spare |
| HEPA | High Efficiency Particle Arrestor |
| IACUC | American [Institutional Animal Care and Use Committee](http://en.wikipedia.org/wiki/Institutional_Animal_Care_and_Use_Committee) |
| ILS | Illumination Subsystem |
| ISS | International Space Station |
| IST | National Cancer Research Institute (Italy) |
| IVC | Individually Ventilated Cages |
| JEM | Japanese Experiment Model |
| JSA | Japanese Space Agency |
| KSC | Kennedy Space Center |
| LED | Light-Emitting Diode |
| LHS | Liquid Handling Subsystem |
| MC | Mice Chamber |
| MDS | Mice Drawer System |
| MGSE | Mechanical Ground Support Equipment |
| NASA | National Aeronautics and Space Administration |
| OSS | Observation Subsystem |
| PCU | Payload Control Unit |
| PI | Principal Investigator |
| PSC | Payload Support Center |
| PTN | Pleiotrophin |
| PTN-Tg | Pleiotrophin transgenic |
| SAS | Space Adaptation Syndrome |
| SLSL | Space Life Science Laboratory |
| SMS | Space Motion Sickness |
| SSPF | Space Station Processing Facility |
| STS | Space Transportation System |
| TSP | Tissue Sharing Program |
| UHB | User Home Base |
| ULF | Utilization and Logistic Flight |
| USOC | User Support Operations Center |
| Wt | Wild type |
